# Supplementary material for: Hot spot prediction in protein-protein interactions by an ensemble system
Source: BMC Syst Biol. 2018 Dec 31;12(Suppl 9):132. doi: 10.1186/s12918-018-0665-8 (PMC6311905; doi:10.1186/s12918-018-0665-8)
Supplement: Supplementary file 2 — All relevant MATLAB code. A simple Matlab implement of our predictor. (ZIP 19,742 kb) [file 12918_2018_665_MOESM2_ESM.zip › Additional file 2/Read ASA/ASA website/dbMPIKT/4NM8B.html]

 NetSurfP - Protein Surface Accessibility and Secondary Structure Predictions

  

|  |  |  |
| --- | --- | --- |
|  |  | NetSurfP - Protein Surface Accessibility and Secondary Structure Predictions  Technical University of Denmark |

  


---

```
# For publication of results, please cite:
# A generic method for assignment of reliability scores applied to solvent accessibility predictions.
# Bent Petersen, Thomas Nordahl Petersen, Pernille Andersen, Morten Nielsen and Claus Lundegaard
# BMC Structural Biology 2009, 9:51 doi:10.1186/1472-6807-9-51
#
# Column 1: Class assignment - B for buried or E for Exposed - Threshold: 25% exposure, but not based on RSA
# Column 2: Amino acid
# Column 3: Sequence name
# Column 4: Amino acid number
# Column 5: Relative Surface Accessibility - RSA
# Column 6: Absolute Surface Accessibility
# Column 7: Z-fit score for RSA prediction
# Column 8: Probability for Alpha-Helix
# Column 9: Probability for Beta-strand
# Column 10: Probability for Coil
E G  4NM8B                  1    0.772  60.741  -1.032   0.016   0.005   0.979
E L  4NM8B                  2    0.298  54.582  -1.136   0.278   0.093   0.628
B F  4NM8B                  3    0.239  48.007  -1.294   0.278   0.093   0.628
B G  4NM8B                  4    0.224  17.621  -1.413   0.428   0.171   0.402
B A  4NM8B                  5    0.186  20.519  -0.751   0.578   0.229   0.194
B I  4NM8B                  6    0.101  18.666   0.254   0.578   0.229   0.194
B A  4NM8B                  7    0.106  11.637  -0.065   0.578   0.229   0.194
B G  4NM8B                  8    0.100   7.886  -0.949   0.578   0.229   0.194
B F  4NM8B                  9    0.090  18.023   0.363   0.538   0.173   0.289
B I  4NM8B                 10    0.094  17.316   0.257   0.428   0.171   0.402
E E  4NM8B                 11    0.342  59.782  -0.421   0.278   0.093   0.628
E N  4NM8B                 12    0.279  40.904  -1.201   0.113   0.043   0.844
B G  4NM8B                 13    0.255  20.045  -1.568   0.058   0.017   0.925
B W  4NM8B                 14    0.093  22.366   0.019   0.184   0.043   0.773
E E  4NM8B                 15    0.396  69.216   0.383   0.278   0.093   0.628
B G  4NM8B                 16    0.099   7.760  -0.996   0.278   0.093   0.628
B M  4NM8B                 17    0.076  15.228   0.320   0.428   0.171   0.402
B I  4NM8B                 18    0.055  10.138  -0.008   0.307   0.165   0.527
B D  4NM8B                 19    0.239  34.498  -1.093   0.307   0.165   0.527
B G  4NM8B                 20    0.097   7.650  -1.288   0.216   0.235   0.548
B W  4NM8B                 21    0.078  18.759  -0.743   0.135   0.317   0.548
B Y  4NM8B                 22    0.151  32.162   0.106   0.135   0.317   0.548
B G  4NM8B                 23    0.073   5.721  -2.298   0.135   0.317   0.548
B F  4NM8B                 24    0.121  24.305   0.246   0.069   0.386   0.545
E R  4NM8B                 25    0.303  69.433   0.373   0.069   0.386   0.545
B H  4NM8B                 26    0.197  35.889  -0.299   0.069   0.386   0.545
E Q  4NM8B                 27    0.447  79.781  -0.263   0.056   0.142   0.802
E N  4NM8B                 28    0.495  72.439  -1.125   0.053   0.043   0.903
E S  4NM8B                 29    0.397  46.575  -1.549   0.053   0.043   0.903
E E  4NM8B                 30    0.509  88.887  -0.745   0.056   0.142   0.802
B G  4NM8B                 31    0.223  17.534  -2.389   0.064   0.216   0.721
E T  4NM8B                 32    0.356  49.419  -0.167   0.135   0.317   0.548
B G  4NM8B                 33    0.174  13.686  -1.981   0.159   0.516   0.325
E Q  4NM8B                 34    0.304  54.294   0.345   0.159   0.516   0.325
B A  4NM8B                 35    0.163  17.996  -1.668   0.231   0.330   0.439
B A  4NM8B                 36    0.273  30.063  -0.541   0.307   0.165   0.527
E D  4NM8B                 37    0.302  43.489  -0.261   0.386   0.097   0.517
E L  4NM8B                 38    0.321  58.684   0.360   0.561   0.047   0.393
E K  4NM8B                 39    0.589 121.096   0.556   0.717   0.014   0.269
B S  4NM8B                 40    0.233  27.308   0.033   0.717   0.014   0.269
B T  4NM8B                 41    0.092  12.816  -0.538   0.879   0.010   0.111
E Q  4NM8B                 42    0.347  62.064   0.656   0.970   0.001   0.030
E A  4NM8B                 43    0.528  58.208   1.368   0.970   0.001   0.030
B A  4NM8B                 44    0.077   8.430  -0.585   0.970   0.001   0.030
B I  4NM8B                 45    0.050   9.268  -0.039   0.970   0.001   0.030
E D  4NM8B                 46    0.497  71.647   0.454   0.970   0.001   0.030
E Q  4NM8B                 47    0.480  85.692   1.036   0.923   0.002   0.076
B I  4NM8B                 48    0.045   8.233  -0.709   0.923   0.002   0.076
B N  4NM8B                 49    0.245  35.883   0.008   0.858   0.002   0.139
E G  4NM8B                 50    0.590  46.433  -0.271   0.858   0.002   0.139
E K  4NM8B                 51    0.365  75.081   0.800   0.858   0.002   0.139
B L  4NM8B                 52    0.053   9.796  -0.175   0.858   0.002   0.139
E N  4NM8B                 53    0.357  52.279  -0.268   0.858   0.002   0.139
E R  4NM8B                 54    0.451 103.210   0.869   0.923   0.002   0.076
B V  4NM8B                 55    0.073  11.282  -0.495   0.938   0.007   0.055
B I  4NM8B                 56    0.070  13.042  -0.032   0.938   0.007   0.055
E E  4NM8B                 57    0.528  92.207   0.570   0.938   0.007   0.055
E K  4NM8B                 58    0.419  86.188   0.801   0.938   0.007   0.055
B T  4NM8B                 59    0.122  16.977  -0.616   0.858   0.002   0.139
E N  4NM8B                 60    0.314  46.028  -0.207   0.782   0.003   0.216
E E  4NM8B                 61    0.626 109.310   0.751   0.858   0.002   0.139
E K  4NM8B                 62    0.398  81.910   0.610   0.802   0.014   0.185
B F  4NM8B                 63    0.087  17.561   0.412   0.879   0.010   0.111
E H  4NM8B                 64    0.546  99.336   0.883   0.802   0.014   0.185
E Q  4NM8B                 65    0.446  79.638   1.185   0.831   0.044   0.125
B I  4NM8B                 66    0.069  12.728   0.334   0.802   0.014   0.185
E E  4NM8B                 67    0.469  81.917   0.545   0.858   0.002   0.139
E K  4NM8B                 68    0.566 116.488   0.221   0.858   0.002   0.139
E E  4NM8B                 69    0.308  53.860   0.176   0.923   0.002   0.076
B F  4NM8B                 70    0.085  17.140  -0.064   0.923   0.002   0.076
E S  4NM8B                 71    0.570  66.757   0.821   0.923   0.002   0.076
E E  4NM8B                 72    0.518  90.512   1.320   0.970   0.001   0.030
B V  4NM8B                 73    0.057   8.822   0.292   0.970   0.001   0.030
B E  4NM8B                 74    0.262  45.806   0.288   0.970   0.001   0.030
E G  4NM8B                 75    0.587  46.236   0.907   0.970   0.001   0.030
B R  4NM8B                 76    0.239  54.662   0.962   0.970   0.001   0.030
B I  4NM8B                 77    0.039   7.160  -0.161   0.970   0.001   0.030
E Q  4NM8B                 78    0.474  84.585   0.781   0.923   0.002   0.076
E D  4NM8B                 79    0.450  64.888   1.062   0.923   0.002   0.076
B L  4NM8B                 80    0.038   6.903  -0.107   0.923   0.002   0.076
E E  4NM8B                 81    0.286  49.999  -0.047   0.858   0.002   0.139
E K  4NM8B                 82    0.628 129.200   0.988   0.858   0.002   0.139
E Y  4NM8B                 83    0.284  60.669   1.035   0.879   0.010   0.111
B V  4NM8B                 84    0.054   8.377   0.328   0.802   0.014   0.185
E E  4NM8B                 85    0.493  86.057   0.231   0.717   0.014   0.269
E D  4NM8B                 86    0.498  71.776  -0.631   0.622   0.015   0.363
B T  4NM8B                 87    0.170  23.551   0.069   0.660   0.049   0.291
B K  4NM8B                 88    0.152  31.246   0.289   0.660   0.049   0.291
B I  4NM8B                 89    0.223  41.255   0.303   0.779   0.100   0.120
B D  4NM8B                 90    0.263  37.841  -0.397   0.779   0.100   0.120
B L  4NM8B                 91    0.057  10.400   0.492   0.779   0.100   0.120
B W  4NM8B                 92    0.074  17.869   0.055   0.779   0.100   0.120
B S  4NM8B                 93    0.157  18.436  -0.465   0.701   0.107   0.192
B Y  4NM8B                 94    0.146  31.286   0.292   0.701   0.107   0.192
B N  4NM8B                 95    0.141  20.686  -0.274   0.660   0.049   0.291
B A  4NM8B                 96    0.052   5.730   0.351   0.751   0.050   0.199
E E  4NM8B                 97    0.250  43.745   0.258   0.831   0.044   0.125
B L  4NM8B                 98    0.039   7.141   0.215   0.870   0.077   0.053
B L  4NM8B                 99    0.051   9.283   0.297   0.870   0.077   0.053
B V  4NM8B                100    0.074  11.451   0.235   0.870   0.077   0.053
B A  4NM8B                101    0.072   7.890  -0.235   0.779   0.100   0.120
B L  4NM8B                102    0.069  12.579   0.138   0.725   0.163   0.112
E E  4NM8B                103    0.344  60.062   0.043   0.386   0.097   0.517
E N  4NM8B                104    0.402  58.780  -0.919   0.113   0.043   0.844
E Q  4NM8B                105    0.463  82.620   0.075   0.053   0.043   0.903
E H  4NM8B                106    0.397  72.269  -0.406   0.056   0.142   0.802
B T  4NM8B                107    0.218  30.195  -0.126   0.064   0.216   0.721
B I  4NM8B                108    0.107  19.869   0.671   0.056   0.142   0.802
E D  4NM8B                109    0.447  64.427   0.187   0.191   0.086   0.723
B L  4NM8B                110    0.292  53.447  -0.335   0.339   0.016   0.645
E T  4NM8B                111    0.440  61.042  -0.671   0.257   0.016   0.727
E D  4NM8B                112    0.331  47.712  -0.565   0.694   0.003   0.303
E S  4NM8B                113    0.577  67.660  -0.391   0.858   0.002   0.139
E E  4NM8B                114    0.403  70.317  -0.058   0.858   0.002   0.139
B M  4NM8B                115    0.069  13.887   0.097   0.923   0.002   0.076
E N  4NM8B                116    0.388  56.745   1.378   0.923   0.002   0.076
E K  4NM8B                117    0.501 102.973   1.290   0.923   0.002   0.076
B L  4NM8B                118    0.063  11.609   0.104   0.970   0.001   0.030
B F  4NM8B                119    0.147  29.603   0.113   0.970   0.001   0.030
E E  4NM8B                120    0.568  99.230   0.993   0.988   0.000   0.012
E K  4NM8B                121    0.413  84.892   1.260   0.988   0.000   0.012
B T  4NM8B                122    0.035   4.882  -0.305   0.970   0.001   0.030
E G  4NM8B                123    0.286  22.500   0.987   0.970   0.001   0.030
E R  4NM8B                124    0.537 122.996   0.927   0.923   0.002   0.076
E Q  4NM8B                125    0.312  55.705   0.824   0.858   0.002   0.139
B L  4NM8B                126    0.059  10.730   0.118   0.694   0.003   0.303
E R  4NM8B                127    0.418  95.814   0.510   0.502   0.002   0.495
E E  4NM8B                128    0.707 123.478   0.134   0.321   0.003   0.675
E N  4NM8B                129    0.398  58.282  -0.533   0.246   0.004   0.750
B A  4NM8B                130    0.118  12.971  -1.711   0.430   0.016   0.555
E E  4NM8B                131    0.516  90.180   0.337   0.660   0.049   0.291
E D  4NM8B                132    0.602  86.734  -0.786   0.751   0.050   0.199
E M  4NM8B                133    0.394  78.919  -0.634   0.522   0.016   0.462
E G  4NM8B                134    0.300  23.602  -1.816   0.257   0.016   0.727
E N  4NM8B                135    0.422  61.781  -0.275   0.115   0.016   0.868
B G  4NM8B                136    0.124   9.751  -0.850   0.113   0.087   0.800
B C  4NM8B                137    0.042   5.883   0.044   0.252   0.423   0.325
B F  4NM8B                138    0.082  16.397   0.674   0.391   0.466   0.143
E K  4NM8B                139    0.318  65.330   0.575   0.487   0.370   0.144
B I  4NM8B                140    0.073  13.505   0.143   0.594   0.285   0.121
B Y  4NM8B                141    0.150  32.012   0.726   0.662   0.225   0.113
B H  4NM8B                142    0.259  47.167   0.131   0.649   0.163   0.188
E K  4NM8B                143    0.465  95.692   1.247   0.502   0.102   0.396
B C  4NM8B                144    0.052   7.231   0.200   0.257   0.016   0.727
E D  4NM8B                145    0.421  60.724   0.276   0.321   0.003   0.675
E N  4NM8B                146    0.658  96.346  -0.310   0.600   0.003   0.397
E A  4NM8B                147    0.527  58.042   1.720   0.782   0.003   0.216
B C  4NM8B                148    0.033   4.605  -0.125   0.879   0.010   0.111
B I  4NM8B                149    0.159  29.471   0.643   0.879   0.010   0.111
E E  4NM8B                150    0.499  87.210   0.530   0.938   0.007   0.055
B S  4NM8B                151    0.260  30.425   0.503   0.879   0.010   0.111
B I  4NM8B                152    0.068  12.525   0.047   0.802   0.014   0.185
E R  4NM8B                153    0.446 102.111  -0.035   0.717   0.014   0.269
E N  4NM8B                154    0.574  84.063  -1.162   0.339   0.016   0.645
E G  4NM8B                155    0.357  28.088  -1.333   0.115   0.016   0.868
E T  4NM8B                156    0.328  45.535  -1.201   0.113   0.043   0.844
B Y  4NM8B                157    0.261  55.861  -0.216   0.113   0.043   0.844
E D  4NM8B                158    0.501  72.266  -0.094   0.115   0.016   0.868
B H  4NM8B                159    0.239  43.420   0.556   0.430   0.016   0.555
E D  4NM8B                160    0.678  97.714   0.359   0.717   0.014   0.269
E V  4NM8B                161    0.470  72.239   1.132   0.717   0.014   0.269
B Y  4NM8B                162    0.129  27.567   0.358   0.717   0.014   0.269
E R  4NM8B                163    0.453 103.737   0.774   0.802   0.014   0.185
E D  4NM8B                164    0.660  95.106   0.179   0.879   0.010   0.111
E E  4NM8B                165    0.446  77.916  -0.094   0.879   0.010   0.111
B A  4NM8B                166    0.131  14.392  -1.030   0.802   0.014   0.185
E L  4NM8B                167    0.450  82.487   0.455   0.717   0.014   0.269
E N  4NM8B                168    0.305  44.608  -0.457   0.717   0.014   0.269
E N  4NM8B                169    0.524  76.670  -0.144   0.522   0.016   0.462
E R  4NM8B                170    0.329  75.295  -0.050   0.561   0.047   0.393
E F  4NM8B                171    0.451  90.476   0.025   0.502   0.102   0.396
E Q  4NM8B                172    0.642 114.643   0.778   0.307   0.165   0.527
B I  4NM8B                173    0.156  28.823  -0.999   0.216   0.235   0.548
E K  4NM8B                174    0.647 133.108  -0.097   0.118   0.150   0.732
E G  4NM8B                175    0.669  52.642  -1.024   0.053   0.043   0.903
E V  4NM8B                176    0.699 107.467  -1.650   0.003   0.003   0.994

Explain the output.  Go back.
```

---
